# Incidence of endophthalmitis after intravitreal injection of an anti-VEGF agent with or without topical antibiotics

**DOI:** 10.1038/s41598-020-79377-w

**Published:** 2020-12-17

**Authors:** Masakazu Morioka, Yoshihiro Takamura, Kazuki Nagai, Shigeo Yoshida, Junya Mori, Masaru Takeuchi, Tomoko Sawada, Kumiko Sone, Hisashi Fukuyama, Sentaro Kusuhara, Tsutomu Yasukawa, Tomoya Murakami, Hitoshi Tabuchi, Daisuke Nagasato, Takao Hirano, Tetsuo Ueda, Tatsuya Jujo, Hirofumi Sasajima, Yoshinori Mitamura, Kunihiro Ishikawa, Masaru Inatani

**Affiliations:** 1 Department of Ophthalmology, Faculty of Medical Sciences, University of Fukui, Yoshida, Japan; 2 Department of Ophthalmology, Gunma University, Maebashi, Japan; 3 Department of Ophthalmology, Kurume University School of Medicine, Kurume, Japan; 4 Department of Ophthalmology, Sapporo City General Hospital, Sapporo, Japan; 5 Department of Ophthalmology, National Defense Medical College, Tokorozawa, Japan; 6 Department of Ophthalmology, Shiga University of Medical Science, Otsu, Japan; 7 Department of Ophthalmology, Tokyo Medical University Hachioji Medical Center, Tokyo, Japan; 8 Department of Ophthalmology, Hyogo College of Medicine, Nishinomiya, Japan; 9 Division of Ophthalmology, Department of Surgery, Kobe University Graduate School of Medicine, Kobe, Japan; 10 Department of Ophthalmology and Visual Science, Nagoya City University Graduate School of Medical Sciences, Nagoya, Japan; 11 Department of Ophthalmology, Faculty of Medicine, University of Tsukuba, Ibaraki, Japan; 12 Department of Ophthalmology, Saneikai Tsukazaki Hospital, Himeji, Japan; 13 Department of Ophthalmology, Shinshu University School of Medicine, Matsumoto, Japan; 14 Department of Ophthalmology, Nara Medical University, Kashihara, Japan; 15 Department of Ophthalmology, St. Marianna University School of Medicine, Kawasaki, Japan; 16 Department of Ophthalmology, Aichi Medical University, Nagakute, Japan; 17 Department of Ophthalmology, Institute of Biomedical Sciences, Tokushima University Graduate School, Tokushima, Japan; 18 Department of Diabetic Ophthalmology, Diabetes Center, Tokyo Women’s Medical University, Tokyo, Japan

**Keywords:** Retinal diseases, Bacterial infection

## Abstract

Intravitreal injection (IVI) of anti-vascular endothelial growth factor (VEGF) is the standard treatment modality in various types of retinal diseases. However, endophthalmitis remains the most serious complication. Despite the lack of evidence that antibiotics prevent endophthalmitis, topical antibiotics are still used routinely in Japan. We conducted a retrospective multicenter study by analyzing records from patients who underwent IVI of anti-VEGF agents with or without antibiotic treatment. In the analysis of a total of 147,440 eyes, the incidence of endophthalmitis was 0.007%: 0.005% with no use of antibiotics, 0.009% with antibiotic pretreatment, 0.012% with posttreatment, and 0.005% with pre- and posttreatment. There was no statistically significant difference among the four groups (chi-square test, *p* = 0.57). Most facilities used masks, sterilized gloves, and drapes. Nine of the 10 eyes that developed endophthalmitis received topical antibiotics, and all infected eyes underwent IVI with aflibercept, not the prefilled syringe delivery system. In four patients who received multiple IVI, the detection of causative bacteria revealed resistance to used antibiotics. Data from this large population, treated with or without antibiotics, suggests that antibiotic prophylaxis does not reduce the rate of endophthalmitis after IVI.

## Introduction

Intravitreal injection (IVI) of anti-vascular endothelial growth factor (VEGF) agents is used widely to treat various retinal diseases, including retinal vein occlusion (RVO), diabetic macular edema (DME), myopic choroidal neovascularization (mCNV), and age-related macular degeneration (AMD). Although anti-VEGF therapy has a promising effect in the treatment of these diseases, multiple injections are required to maintain its therapeutic effect. Two types of VEGF inhibitor are approved, namely, aflibercept and ranibizumab, and the number of IVIs annually performed has increased significantly^1^.

The most serious complication after IVI is endophthalmitis, which is rare, with an incidence ranging from 0.01 to 0.26%^2^. Delayed treatment of endophthalmitis may cause blindness. US guidelines state that applying povidone-iodine (PI) to the ocular surface before the injection can prevent endophthalmitis^3^. The efficacy of antibiotics for prophylaxis has not been proven clinically. Recent meta-analyses have shown that antibiotic prophylaxis does not reduce the incidence of endophthalmitis after IVI^2,4–7^. Moreover, frequent exposure to antibiotic eye drops increases the antibiotic resistance of ocular surface flora and may result in a higher risk of endophthalmitis. In fact, in some cases, the rates of endophthalmitis are higher in eyes that have received repeated and short-term antibiotics^8,9^.

Although the trend appears to be not using antibiotics before or after IVI, in some geographic areas, including Japan, topical antibiotics are still used routinely. In a survey of Japanese ophthalmologists, 97.2% of respondents used antibiotics before and/or after IVI, whereas only 2.8% reported not using topical antibiotic eye drops^10^. The Japanese Retina and Vitreous Society states the choice to use antibiotics related to IVI is at the discretion of ophthalmologists^11^. However, the package inserts of ranibizumab and aflibercept recommend using topical antibiotics before and after injections for one week per injection, which greatly influences clinicians’ preferences. A large-scale research study in Japan, where antibiotic agents are widely used, will provide useful information for better understanding their effect on the prevention of infections in IVI. Because low incidence of endophthalmitis require the large sample size, we collected a large amount of data from multiple centers in Japan.

## Results

### Questionnaire

All 18 facilities participating in the study responded to the questionnaire. The results of the questionnaire were tabulated, and each response was calculated as a percentage of the total (Table 1). In all facilities, the conjunctival sac was washed with iodine compound after eye speculum placement, then injection was performedin all cases; 12 (66.7%) and 6 (33.3%) facilities used polyvinyl alcohol-iodine (PAI) and PI, respectively. The rates of using drapes, sterile gloves, and masks were 15 (83.3%), 18 (100%), and 17 (94.4%), respectively. In three facilities in which drapes were not used, the patients wore masks instead. IVI was carried out in the outpatient room in 15 (83.3%) facilities and the operation room in three facilities.

**Table 1:** The results of the questionnaire.

Question	Answer	Use of antibiotics based on Q1 (no. of facilities)			
		None (3)	Preinjection only (1)	Postinjection only (5)	Preinjection and postinjection (9)
2. What kind of antibiotics do you use?	A:Fluoroquinolone B:Others	–	100 [1/1]	100 [5/5]	100 [9/9]
3. What do you use for conjunctival sac disinfection?	A:PA Iodine B: Povidone Iodine	100 [3/3]	100 [1/1]	60 [3/5]	55 [5/9]
4. Do you use sterile drapes?	A:Yes B:No	100 [3/3]	100 [1/1]	60 [3/5]	88 [8/9]
5. Does the operator wear sterile groves?	A:Yes B:No	100 [3/3]	100 [1/1]	100 [5/5]	100 [9/9]
6. Does the operator wear a mask?	A:Yes B:No	100 [3/3]	100 [1/1]	80 [4/5]	100 [9/9]
7. Does the patient wear a mask?	A:Yes B:No	0 [0/3]	0 [0/1]	40 [2/5]	11 [1/9]
8. Does the operator wear a cap?	A:Yes B:No	100 [3/3]	100 [1/1]	60 [3/5]	44 [4/9]
9. Where do you perform intravitreal injections?	A: outpatient room B: operation room	66 [2/3]	0 [0/1]	80 [4/5]	100 [9/9]

The number shows the percentage of answer A.

### Incidence rate of endophthalmitis

A total of 147,440 IVIs were performed, and 10 patients developed endophthalmitis. Thus, the incidence rate was 0.007% (95% confidence interval [CI] 0.00368–0.0125%).

Among 18 facilities, 3 (16.7%) did not use any topical antibiotics before or after IVIs. One facility (5.6%) used antibiotics only before IVI for three days, four facilities (22.2%) used antibiotics only after IVI for three days, and 10 facilities (55.6%) used antibiotics both pre- and postinjection for seven days. Patients were instructed to use eye drops three times a day.

Table 2 shows the incidence of endophthalmitis in the four groups as defined by the use of topical antibiotics. The rate was 0.005% (95% CI 0.000894–0.0287%) with no antibiotic use, 0.009% (95% CI 0.00162–0.0519%) with pretreatment antibiotics, 0.012% (95% CI 0.00465–0.0308%) with posttreatment antibiotics, and 0.005% (95% CI 0.00187–0.0125%) with pre- and posttreatment antibiotics. There was no statistically significant difference in the incidence rates among the four groups (chi-square test, *p* = 0.57).

**Table 2:** The incidence of endophthalmitis in the four groups as defined by the use of topical antibiotics.

Antibiotic use	No. of facilities	No. of injections	No. of endophthalmitis	Incident rate (%)	95% Confidence interval
None	3	19,738	1	0.005	0.000894–0.0287%
Preinjection only	1	10,903	1	0.009	0.00162–0.0519%
Postinjection only	4	33,433	4	0.012	0.00465–0.0308%
Pre- and postinjection	10	83,366	4	0.005	0.00187–0.0123%
Total	18	147,440	10	0.007	0.00368–0.0125%

Table 3 displays the incidence of endophthalmitis in patients with the four diseases (AMD, DME, RVO, and mCNV) who were treated by IVI with ranibizumab or aflibercept. The rates were 0.005% (95% CI 0.00228–0.0125%) in AMD, 0.004% (95% CI 0.000685–0.0220%) in RVO, 0.025% (95% CI 0.00436–0.140%) in mCNV, and 0.012% (95% CI 0.00423–0.0366%) in DME. There was no statistically significant difference in the incidence rates among the diseases (chi-square test, *p* = 0.30). The rate in the eyes that received an IVI of aflibercept was 0.01% (95% CI 0.0052–0.0176%), while there was no eyes of endophthalmitis after injection of ranibizumab. There was statistically significant difference between them (chi-square test, p = 0.041).

**Table 3:** The incidence of endophthalmitis in patients with the four diseases.

Disease	Anti-VEGF agent	None	Preinjection	Postinjection	Pre- and postinjection
AMD	Ranibizumab	0/2336	0/2635	0/3638	0/11,918
	Aflibercept	1/11,123	0/2766	3/17,504	1/41,596
RVO	Ranibizumab	0/1576	0/1637	0/3122	0/5722
	Aflibercept	0/1636	1/563	0/3931	0/7574
mCNV	Ranibizumab	0/341	0/20	0/424	0/1141
	Aflibercept	0/115	0/3	0/362	1/1646
DME	Ranibizumab	0/1308	0/2132	0/1169	0/4021
	Aflibercept	0/1303	0/1147	1/3283	2/9748

Table 4 presents the characteristics of the 10 patients who suffered from endophthalmitis. Only one patient did not receive topical antibiotics. After receiving a diagnosis of endophthalmitis, all patients underwent vitrectomy and IVI of antibiotics. Among them, 8 patients showed positive culture results. Four of the eight patients showed resistance to used antibiotics, all of whom had a history of multiple injections. In one case, *Staphylococcus aureus* was detected at the 13th injection. In three cases, *Staphylococcus epidermidis* was detected at the 9th, 6th, and 2nd injections, respectively. Only one patient had endophthalmitis after the first injection. In terms of visual acuity, four patients recovered to their preinjection level, and significant visual impairment remained in six patients.

**Table 4:** The characteristics of the 10 patients who suffered from endophthalmitis.

Patient No	Agent	Disease	Antibiotic use	Visual acuity (logMAR)			Culture results	Resistance to used antibiotics	No. of injections
				At injection	At presentation	At 3 months after endophthalmitis			
1	Aflibercept	AMD	None	0.70	CF	0.70	*Staphylococcus capitis*	Not tested	5
2	Aflibercept	RVO	Preinjection only	0.00	HM	NLP	*Klebsiella pneumonie*	Not tested	1
3	Aflibercept	AMD	Postinjection only	0.22	CF	0.82	*Staphylococcus aureus*	Yes	13
4	Aflibercept	AMD	Postinjection only	0.00	1.70	0.05	Negative	–	18
5	Aflibercept	DME	Postinjection only	0.52	HM	1.10	*Staphylococcus epidermidis*	Yes	9
6	Aflibercept	AMD	Postinjection only	0.00	HM	0.40	*Staphylococcus epidermidis*	Yes	6
7	Aflibercept	DME	Preinjeciton and postinjection	0.30	CF	0.70	Negative	–	5
8	Aflibercept	DME	Preinjeciton and postinjection	1.00	1.30	1.00	*Staphylococcus epidermidis*	Yes	2
9	Aflibercept	AMD	Preinjeciton and postinjection	0.70	CF	0.70	*Staphylococcus capitis*	Not tested	5
10	Aflibercept	mCNV	Preinjeciton and postinjection	0.82	LP	HM	*Enterococcus faecalis*	No	2

*HM* hand motion, *CF* counting finger, *LP* light perception, *NLP* no light perception.

## Discussion

In Japan, the guidelines for IVI for macular diseases stipulate that the need for antibiotic eye drops in patients undergoing IVI should be determined individually by each facility or practitioner^11^. Thus, we collected and compared the frequency data of endophthalmitis after IVI with and without instilling antibiotics. Similar to the results of several meta-analyses, our data demonstrated that there was no significant difference in the incidence of endophthalmitis between the eyes that did and did not receive a topical application of antibiotic eye drops^2,4–7^. Furthermore, there was no significant difference in endophthalmitis rates among the eyes that received antibiotics at different times (i.e., before, after, or both before and after injection). Based on our data, the topical use of antibiotics did not affect the inhibition of endophthalmitis.

In this study, the overall incidence of endophthalmitis (0.007%) was lower than that previously reported in the United States (from 0.016 to 0.053%)^12^. One reason may be that the US guidelines only recommend using sterile gloves^3^, but the Japanese guidelines clearly state that they should be used^11^. Following the results of the questionnaire survey, most facilities used gloves, masks, and drapes, all of which improve cleanliness during the injection. Nevertheless, the onset of endophthalmitis could not be prevented completely. Preoperative irrigation of eyelid margins and conjunctiva with PI, and avoiding touching the eyelid, may also be important during injection procedures.

Topical PI is currently the only evidence-based method for reducing the incidence of endophthalmitis after IVI^5^. Moss et al. assessed the efficacy of PI in combination with topical antibiotics for three days before the injection^13^. Antibiotic pretreatment resulted in a significantly lower frequency of conjunctival bacterial growth before the administration of topical PI; however, after the administration of topical PI, the rate of positive bacterial culture in antibiotic-treated eyes and untreated controls (PI alone) was similar. This finding supports topical PI as a sufficient tool for infection prevention in the IVI setting. In Japan, the use of a diluted PAI solution in preoperative eyewash is approved. In our data, PAI or PI was used as an iodine compound for washing the conjunctival sac. Inoue et al. reported that PAI was not inferior to PI as a preoperative disinfectant^14^.

Because IVI of an anti-VEGF agent is frequently performed, antibiotic eye drops will also be repeated. Repeated use of antibiotic eye drops has reportedly increases the percentage of drug-resistant bacteria and to alter the proportion of bacteria that compose the bacterial flora^15–17^. Conversely, studies indicate that without the use of antibiotic eye drops, repeated IVI did not change the bacterial flora^18^. These findings support that recommendation against the routine use of the topical antibiotics for IVI prophylaxis. Concerning the causative organisms detected in our data, four cases were resistant to antibiotics and had a history of multiple IVIs. All participating facilities commonly used fluoroquinolones, which have a broad spectrum and high penetration into the tissues. Some studies have reported substantial levels of resistance to third- and fourth-generation fluoroquinolones in patients treated with topical antibiotics after multiple IVIs^19,20^. In our study, all four eyes with endophthalmitis due to antibiotic resistant bacteria underwent the topical application of antibiotics. The use of repeated topical antibiotics may result in antibiotic-resistant bacteria.

Our data showed that a common issue in all cases of endophthalmitis was the use of aflibercept. Conversely, studies report that the incidence of endophthalmitis was similar after the administration of three different anti-VEGF agents: bevacizumab, 0.013%; ranibizumab, 0.016%; and aflibercept, 0.016%.^1^ The reason for this discrepancy might be that, currently, ranibizumab is used with prefilled syringes, whereas aflibercept has a conventional preparation. Indeed, Storey et al. reported that the use of prefilled syringes during IVI of ranibizumab was associated with a reduced rate of culture-positive endophthalmitis^21^. With the conventional preparation, there may be a risk of contamination when transferring the drug solution from the bottle to the syringe.

Following the habits of cataract surgery, in Japan, the package insert of anti-VEGF agents clarifies the necessity of antibiotic instillation for three days before and after IVI. Because the package insert is a formal document that can be used for legal purposes, many ophthalmologists follow the description of the package insert in consideration of litigation risk. However, our data and the findings of recent studies demonstrated that antibiotics offer no protection against the risk of developing endophthalmitis after administration of anti-VEGF agents. The repeated application of antibiotics with multiple injections may lead to an increase in antibiotic resistance. It is also important to note that not administering antibiotics before and after IVI leads to significant cost savings. Cleaning the eyelid margins and conjunctiva with an iodine compound and avoiding touching the needle are essential practices. Also, ophthalmologists, especially in Japan, should recognize that using antibiotics in IVI of anti-VEGF agents is unnecessary and even possibly harmful to patients.

Due to the nature of the retrospective and multi-center study, there are several biases that other factors than antibiotics used are different among facilities. It is possible that 3 institutes of no antibiotics have established systems and the way to perform injections to prevent endophthalmitis. However, the result of questionnaire showed that the difference of background among facilities was small to influence on the ratio of endophthalmitis.

## Methods

We collected data from 18 clinical centers throughout Japan. the University of Fukui Institutional Review Board and the ethics committees of the other participating facilities (Gunma University, Kurume University, Sapporo City General Hospital, National Defense Medical College, Shiga University, Tokyo Medical University Hachioji Medical Center, Hyogo College of Medicine, Kobe University, Nagoya City University, University of Tsukuba, Saneikai Tsukazaki Hospital, Shinshu University, Nara Medical University, St. Marianna University, Aichi Medical University, Tokushima University, and Tokyo Women's Medical University) approved the study protocol. All study procedures adhered to the tenets established by the Declaration of Helsinki. Informed consent was obtained from all participants. We retrospectively reviewed the medical records of patients who underwent IVI of ranibizumab, aflibercept, and TA between January 1, 2015, and December 31, 2019, for the treatment of AMD, mCNV, RVO, and DME. The cultures were incubated in automated microbiology systems. On the basis of automated readings, antibiotic sensitivity using the categories of susceptible, intermediate, and resistant were determined.

IVIs were performed in a conventional manner by a trained ophthalmologist using 0.4% oxybuprocaine hydrochloride (0.4% benoxyl ophthalmic solution; Santen Co. Ltd., Osaka, Japan) as an anesthetic. An eyelid speculum was used to stabilize the eyelid. The injection volumes of ranibizumab (Lucentis; Novartis Pharma KK, Tokyo, Japan) and aflibercept (Eylea; Bayer Yakuhin, Ltd. Tokyo, Japan) were 0.5 mg/0.05 mL and 2 mg/0.05 mL, respectively.

A questionnaire was administered in each facility to obtain background information on the use of injections and prophylaxis for infection. The questions were as follows: (1) How do you use topical antibiotics? (2) If yes, what kinds of antibiotics do you use? (3) What do you use for conjunctival sac disinfection? (4) Do you use sterile drapes? (5) Does the operator wear sterile groves? (6) Does the operator wear masks? (7) Does the patient wear masks? (8) Does the operator wear a cap? (9) Where do you perform IVIs?

Statistical analyses were performed using JMP (SAS Institute Inc., Tokyo, Japan). The incidence rates and associated 95% CIs were calculated on a per-injection basis. The chi-square test was used to assess the difference between groups defined by the use of antibiotics and injected agents. The level of statistical significance was set as *p* < 0.05.

## Data Availability

The data sets generated during and/or analyzed during the current study are available from the corresponding author on reasonable request.
